# Novel frameshift variant of *WNT10A* in a Japanese patient with hypodontia

**DOI:** 10.1038/s41439-023-00259-4

**Published:** 2024-01-23

**Authors:** Michiyo Ando, Yoshihiko Aoki, Yasuto Sano, Junya Adachi, Masatoshi Sana, Satoru Miyabe, Satoshi Watanabe, Shogo Hasegawa, Hitoshi Miyachi, Junichiro Machida, Mitsuo Goto, Yoshihito Tokita

**Affiliations:** 1 Department of Maxillofacial Surgery, School of Dentistry, Aichi-Gakuin University, Nagoya, Japan; 2 Department of Disease Model, Institute for Developmental Research, Aichi Developmental Disability Center, Kasugai, Japan; 3 Department of Oral and Maxillofacial Surgery, Okazaki Municipal Hospital, Okazaki, Japan; 4 Department of Oral and Maxillofacial Surgery, Toyohashi Municipal Hospital, Toyohashi, Japan; 5 Nagoya Orthodontic Clinic, Nagoya, Japan; 6 Department of Oral and Maxillofacial Surgery, Toyota Memorial Hospital, Toyota, Japan

**Keywords:** Rare variants, Mutation

## Abstract

Congenital tooth agenesis is caused by the impairment of crucial genes related to tooth development, such as Wnt signaling pathway genes. Here, we investigated the genetic causes of sporadic congenital tooth agenesis. Exome sequencing, followed by Sanger sequencing, identified a novel single-nucleotide deletion in *WNT10A* (NC_000002.12(NM_025216.3):c.802del), which was not found in the healthy parents of the patient. Thus, we concluded that the variant was the genetic cause of the patient’s agenesis.

The Wingless-related integration site (WNT) ligand family comprises 19 highly conserved genes across species, ranging from invertebrates to mammals^1^. These genes encode secreted glycoproteins linked to the canonical intracellular beta-catenin signaling pathway, which plays a critical role in multiple tissues and organ development. One of the family members, WNT10A, is known to have specific relevance to the skin, skin appendages, and teeth^2^. Pathogenic variants in the *WNT10A* gene are the most frequent cause of nonsyndromic tooth agenesis in humans, including the Japanese population^3^.

Congenital tooth agenesis is a condition characterized by missing teeth, which can vary in number and type. It ranges from selective tooth agenesis to syndromic conditions such as ectodermal dysplasia^4^. Nonsyndromic hypodontia, a mild selective tooth agenesis defined as missing fewer than six teeth, is a common congenital disorder in humans. A more severe phenotype, oligodontia, involves the loss of six or more permanent teeth. In the Japanese population, the frequency is 6.8% for hypodontia and 0.1% for oligodontia^3^. The etiology of congenital tooth agenesis has been investigated, and several causative genes have been identified^5–12^. Many genes have been reported as etiologic agents of tooth agenesis, including *MSX1*, *PAX9*, *LRP6*, *WNT10A*, and *WNT10B*^13^. Recent studies have highlighted *WNT10A* as a significant causative gene with diverse effects on gene/protein function^14–18^. According to genetic studies on families with tooth agenesis, the *WNT10A* pathogenic variant is the most frequent cause of human tooth agenesis (STHAG4, OMIM:150400)^17^.

Here, we report the clinical genetic analysis of a patient with a sporadic form of nonsyndromic hypodontia with three congenitally missing teeth.

Saliva samples were obtained from the proband (II-1), her unaffected siblings (II-2), and both parents (I-1 and I-2; Fig. 1a). The patient’s parents provided written informed consent. The Institute for Developmental Research and the Aichi-Gakuin University Committee approved this clinical and molecular genetic study, which was conducted in accordance with the Declaration of Helsinki. The patient (II-1; Fig. 1a) was a 7-year-old girl who presented with missing teeth 17, 24, and 27 (Fédération Dentaire International tooth numbering system). Orthopantomography confirmed a missing tooth in the mandible (Fig. 1b). The patient had no systemic abnormalities except for tooth number, including the crown morphology of the other teeth or the jawbone. Furthermore, no abnormalities in tooth number were found in the other family members (Fig. 1a).

**Fig. 1:**
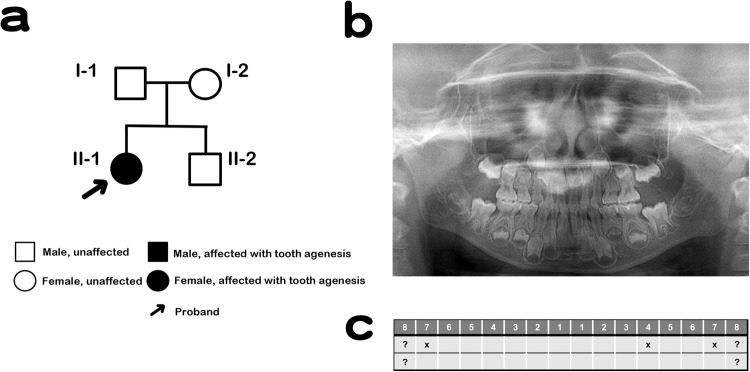
Pedigree diagram showing the segregation of the WNT10A variant in an autosomal dominant manner. **a** Male and female members are shown by squares and circles, respectively. The filled circle denotes the affected participant. **b** Phenotypic characteristics of the patient. X-ray imaging of the proband. **c** X indicates the missing teeth of the patient.

According to the manufacturer’s protocol, the Oragene DISCOVER kit was used to extract genomic DNA from 2 ml of saliva. Whole-exome sequencing was performed using a SureSelect Human All Exon Kit (Agilent Technologies, Santa Clara, CA, USA), and the captured libraries were sequenced using an Illumina NovaSeq 6000 (Illumina, San Diego, CA, USA) with 150 base pair paired-end reads. Whole-exome sequencing (WES) of the patient’s genomic DNA identified a *WNT10A* variant, NC_000002.12(NM_025216.3):c.802del, in the proband (II:1). Trio-based Sanger sequencing with a specific primer set (5’-CTCAGCGTTTGCCTCTGTA-3,’ 5’-ACGAAACAGCACCAGTGGAA-3’) confirmed that this was a de novo variant (Fig. 2). This variant is not found in the online gnomAD database (https://gnomad.broadinstitute.org/). According to the ACMG-AMP Guidelines (PVS1 and PS2), the variant was classified as pathogenic.

**Fig. 2:**
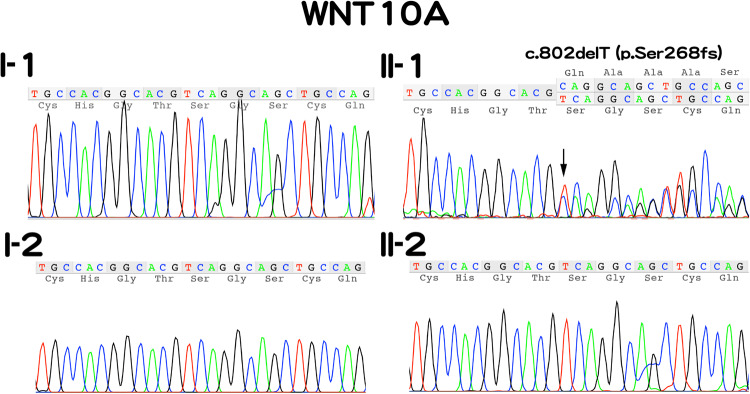
Electropherograms of Sanger sequencing of the WNT10A gene of all members of the family. The Sanger sequencing chromatogram demonstrated heterozygosity for the *WNT10A* variant (c.802delT) in the proband (II-1).

The C-terminal region of the WNT ligand plays a pivotal role in binding to FRIZZLED (FZD), a WNT receptor with seven transmembrane domains. The *WNT10A* variant in the current case, p.Ser268fs, would result in a loss of FZD binding activity, similar to other variants included in our previous report^19^. WNT ligands, including *WNT10A*, are cysteine-rich morphogens that can interact with the FZD receptor and LDL receptor-related protein 5/6 (*LRP5/6*). The nucleotide substitution identified in the current case resulted in a frameshift at nucleotide 802. Hence, the *WNT10A* gene variant product had an unrelated peptide consisting of 11 amino acid residues, QAAASSRRAGRX, after the 268th Lys at the C-terminus: NM_025216.3(NP_079492.2):p.(Ser268Glnfs*12). Thus, the variant product lacks a functional domain that interacts with the WNT receptors of FZD.

Although functional null variants of the *WNT10A* gene cause autosomal recessive ectodermal dysplasia, patients with a heterozygous null variant of the *WNT10A* gene are often diagnosed with nonsyndromic tooth agenesis but rarely with mild ectodermal dysplasia. This is because slight anomalies in other ectodermal tissues are more difficult to detect than those related to the number of teeth.

The number of missing teeth varies among patients, even among family members carrying the same variant^14,20^. Therefore, while the *WNT10A* variant caused hypodontia in the current patient, the same variant can also cause oligodontia. The molecular mechanisms underlying the phenotypic variation in tooth number among patients carrying identical gene variants should be elucidated in the future.

## HGV database

The relevant data from this Data Report are hosted at the Human Genome Variation Database at 10.6084/m9.figshare.hgv.3345.
